# Diagnostic Workup of Neonates With Esophageal Atresia: Results From the EUPSA Esophageal Atresia Registry

**DOI:** 10.3389/fped.2020.00489

**Published:** 2020-08-25

**Authors:** Francesco Morini, Andrea Conforti, Augusto Zani, Sanja Sindjic-Antunovic, Antti Koivusalo, Florian Friedmacher, Ernest van Heurn, Agostino Pierro, Michael Hollwarth, Pietro Bagolan

**Affiliations:** ^1^Ospedale Pediatrico Bambino Gesù, IRCCS, Rome, Italy; ^2^The Hospital for Sick Children, Toronto, ON, Canada; ^3^University Children's Hospital, University of Belgrade, Belgrade, Serbia; ^4^Helsinki University Central Hospital, Helsinki, Finland; ^5^Our Lady's Children's Hospital, Dublin, Ireland; ^6^Paediatric Surgical Center Amsterdam, Emma Children's Hospital/VU Medical Center, Amsterdam, Netherlands; ^7^Department of Pediatric and Adolescent Surgery, Medical University of Graz, Graz, Austria

**Keywords:** esophageal atresia, registry analysis, tracheobronchoscopy, preoperative, diagnostic work up

## Abstract

**Aim:** Controversies exist on the optimal diagnostic workup for neonates with esophageal atresia (EA) with/without tracheoesophageal fistula (TEF). Aim of this study was to describe the current diagnostic policies in EA/TEF patients enrolled in an International multicenter registry.

**Methods:** All patients consecutively registered from July 2014 to December 2017 in the EUPSA Esophageal Atresia Registry (EUPSA-EAR) were included in the study. Data related to diagnostic investigations among Centers forming the EUPSA-EAR were analyzed.

**Main Results:** During the study period, 374 consecutive patients were recorded by 23 Centers. The majority of patients underwent chest X-rays, echocardiography, abdominal ultrasound, and abdominal X-rays. Preoperative bronchoscopy and esophageal gap measurement were performed in one third of the patients.

**Conclusions:** Present data from a large cohort of patients from the EUPSA-EAR show both inter-institutional and intra-institutional variability in diagnostic workup of patients with EA/TEF. Efforts should be made to develop guidelines on the diagnostic workup for EA/TEF patients.

## Introduction

In 1974, Myers described esophageal atresia (EA) with/without tracheoesophageal fistula (TEF) as “the epitome of modern surgery” (1) representing the compendium of all distinct features of pediatric surgery. With such a binding definition, it may be expected that four decades later the management of EA/TEF was well-defined and coded. However, recent studies exploring the practice patterns in different centers have shown that controversies still exist in several aspects of EA/TEF management (2–5). Most of the recent studies that collected multicenter data on the management of patients with EA/TEF focused predominantly on operative approach and outcomes (2–8). Interestingly, less attention has been dedicated to EA/TEF diagnostic work-up, although this is crucial to drive the most appropriate treatment and to detect associated conditions that may have a substantial impact on patient outcomes. The aim of this study was to analyze the current diagnostic policies in EA/TEF patients enrolled in the first international multicenter registry dedicated to EA/TEF and to describe inter-institutional and intra-institutional variability in such policies.

## Methods

The European Paediatric Surgeons' Association Esophageal Atresia Registry (EUPSA-EAR; http://eupsa-registry.org/registry) is a voluntary international collaboration born in 2014 to collect data on infants with EA/TEF managed at participating Institutions. The EUPSA-EAR is based in the University of Graz and has been approved for use by its Ethical Committee (number 27–259 ex 14/15). It is constituted of pediatric surgical units from different European and non-European countries, whose Heads are EUPSA members, and has two aims: to allow individual centers to compare their results with the other participating centers for benchmarking and to promote retrospective research studies that would provide the basis for prospective studies. Data on all infants with EA who were born at or transferred to a participating center before any major esophageal surgery were collected anonymously with an online form, cross-checked for congruity, and entered into a central registry database. Data collected prospectively contained information on prenatal period and postnatal hospital care (including surgery when applicable) until death or hospital discharge, and included data on demographics, pre-operative, operative, and post-operative management received, and outcomes. For the present study, we considered the diagnostic work-up of all patients with EA/TEF entered in the EUPSA-EAR between July 2014 and July 2017. All recorded diagnostic procedures, including chest, abdominal or skeletal X-rays, cardiac, abdominal or head ultrasounds, preoperative bronchoscopy, esophagoscopy and gap assessment, and chromosomal investigations were noted and differences in their use within and between participating centers analyzed. Individual research studies using EUPSA-EAR data do not require further separate ethical approval, if only anonymized data are used.

### Statistical Analysis

Data were analyzed using IBM SPSS Package version 21 (IBM Corporation, Armonk, NY, USA; https://www.ibm.com/products/spss-statistics). Chi-square test or Fisher's exact test were used to compare the prevalence of each diagnostic study in the participating centers. Pearson's test was used to analyze the correlation between number of patients entered in the database from each Institution and prevalence of each diagnostic study. Probability values <0.05 were considered significant and two-sided *p-*values are reported.

Results are reported as prevalence and median (range).

## Results

**Figure 1:**
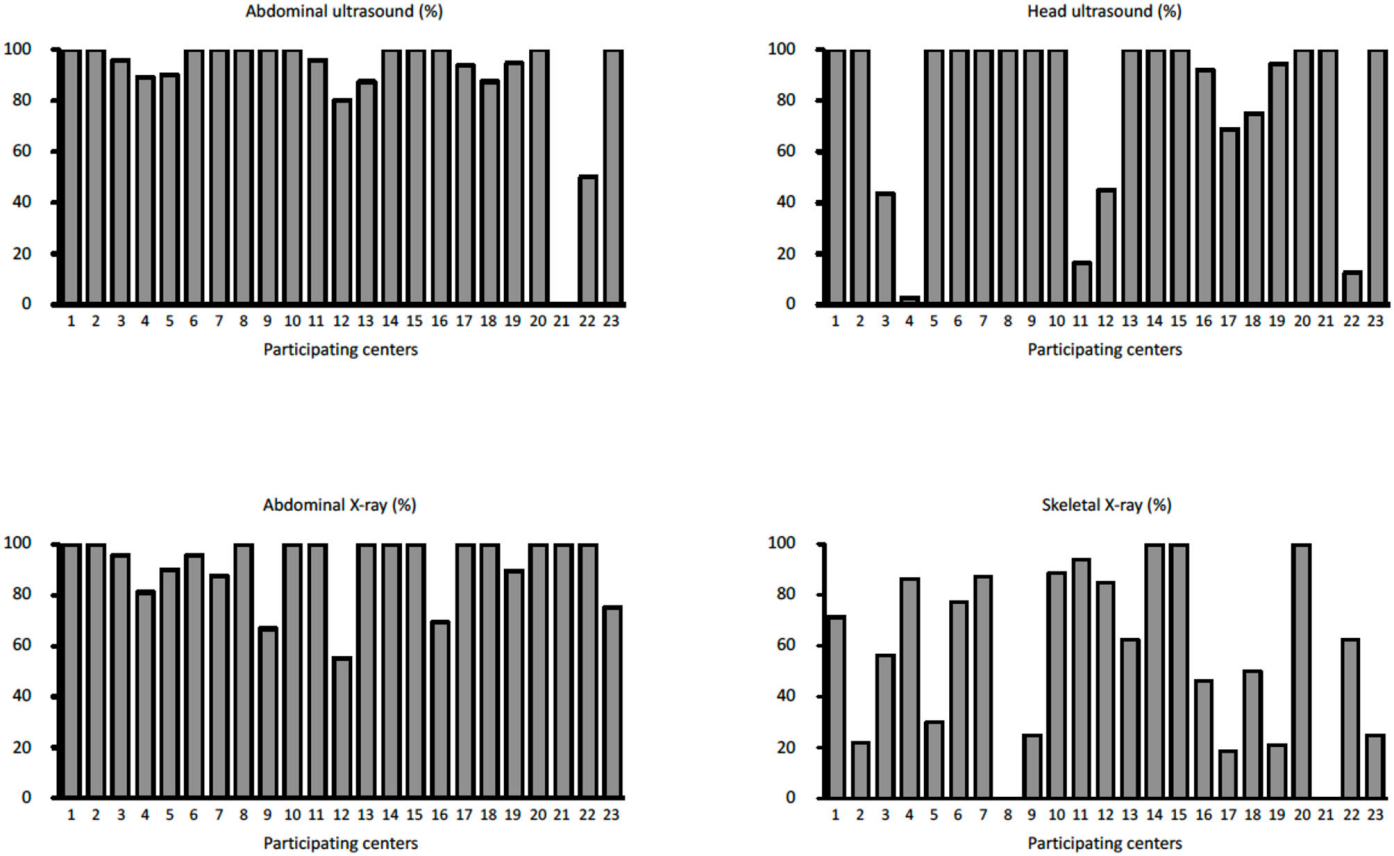
Prevalence of abdominal ultrasound, head ultrasound, abdominal x-rays, and skeletal x-rays in each Institution participating in the EUPSA-EAR.

**Figure 2:**
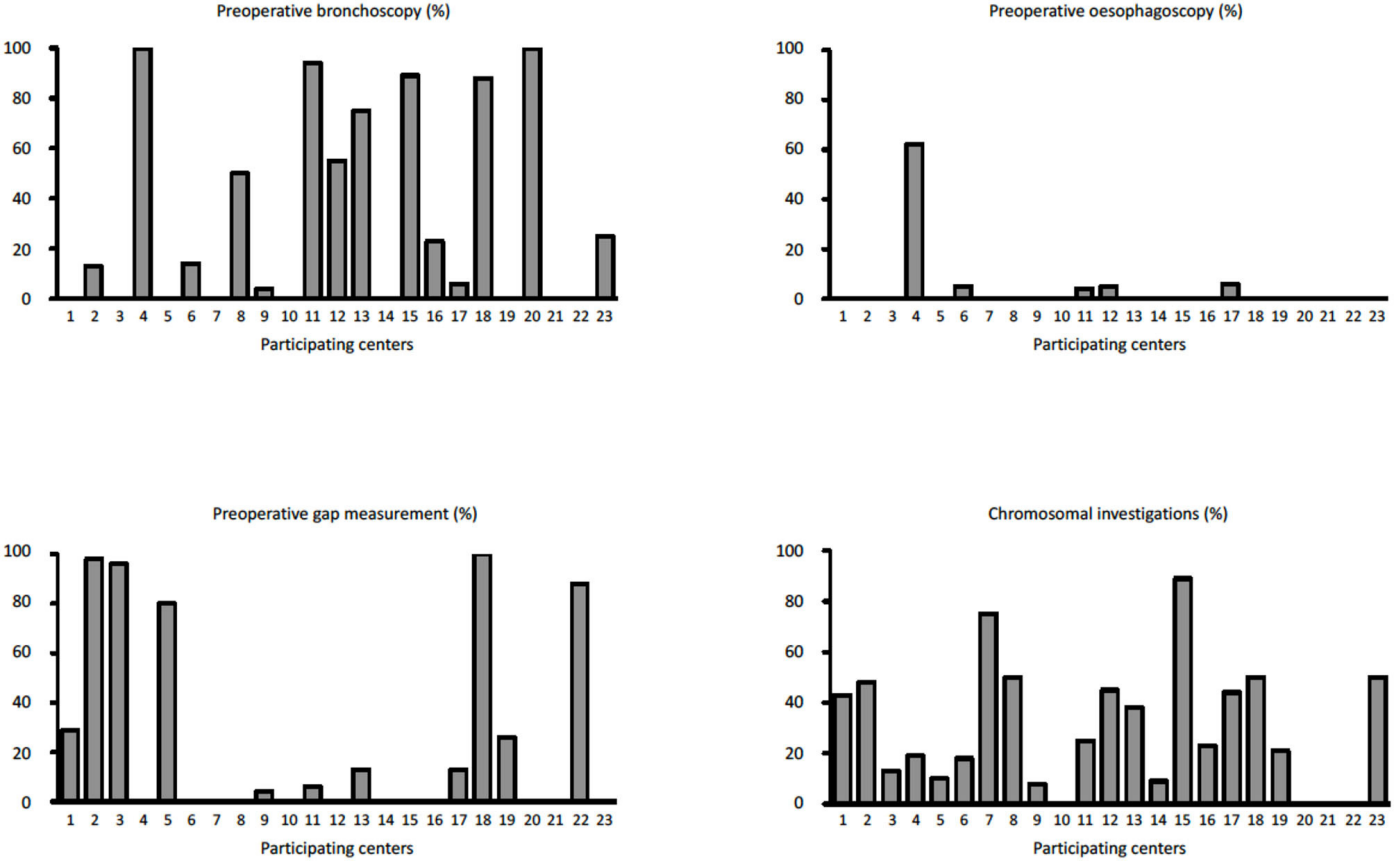
Prevalence of pre-operative bronchoscopy, esophagoscopy, pre-operative gap assessment, and chromosomal investigations in each Institution participating in the EUPSA-EAR.

During the study period, 23 centers provided data on 374 neonates with EA/TEF (median number of patients per center 18, range 1–54; Table 1). Twenty-eight (8%) patients died. Table 2 summarizes demographic data of patients' population. Table 3 shows main findings. Chest X-ray and echocardiography were almost uniformly performed in all patients, while esophagoscopy was reported in only 8% of patients, being the less performed diagnostic procedure. Bronchoscopy was performed in 137 (37%) patients. In particular, only 14% of the patients without a distal TEF had a bronchoscopy. The gap was measured pre-operatively in 112 (30%) patients. The methods for gap measurement included (more than one possible) X-ray (105 patients), bronchoscopy (47 patients), and bronchoscopy and a rigid instrument (endoscope/hegar dilator; 19 patients). Except for chest x-ray and echocardiogram, all the other diagnostic procedures were performed with some variability among the participating centers (Figures 1, 2). No correlation was found between the number of patients entered in the database by the Institutions and the prevalence of each diagnostic study included (Table 4). Fifteen patients entered in the Registry had the most rare forms of EA (6 type D, 5 type B, 4 type E according to Gross classification (9)). The sub-analysis of these patients shows that also these rarest forms were studied in variable way. All had cardiac ultrasound, all but one had an abdominal ultrasound, abdominal X-rays were performed in 12 patients, head ultrasound in 11, skeletal x-rays in 9, only 4 had a bronchoscopy reported, 2 had an esophagoscopy, the gap was measured in 2 and chromosomal study was performed in 3.

**Table 1:** Number of patients in each participating Institution.

**Center id**	**No. of patients**
1	7
2	54
3	23
4	37
5	10
6	22
7	16
8	2
9	24
10	9
11	49
12	20
13	8
14	11
15	9
16	13
17	16
18	8
19	19
20	4
21	1
22	4
23	8

**Table 2:** Demographic data of the study population.

**Variable**	**Median**	**IQ Range**
Gestational age (weeks)	38	35–39
Birth weight (grams)	2,670	2,054–3,150
Age at surgery (days)	2	2–3
**Type of EA (Gross)**		**Pts (%)**
A		30 (8%)
B		5 (1%)
C		329 (88%)
D		6 (2%)
E		4 (1%)
**Prenatal diagnosis**		90 (24%)
**Associated anomalies**		**Pts**
Cardiac		169
Renal		55
Musculoskeletal		71
Anorectal		44
Other		21

*Associated anomalies are not expressed as % as one patient may have more than one, even in the same group (e.g., two or more cardiac anomalies in one single patient)*.

**Table 3:** Prevalence of different diagnostic investigations in the participating Institutions.

**Study**	**Patients (%)**	**Median %**	**IQ Range %**	***p***
Chest X-ray	372 (99%)	100	(100–100)	ns
Echocardiography	365 (98%)	100	(100–100)	ns
Abdominal US	353 (94%)	100	(90–100)	0.0001
Abdominal X-ray	338 (90%)	100	(90–100)	0.0001
Head US	257 (69%)	100	(73–100)	0.0001
Skeletal X-ray	225 (60%)	63	(25–88)	0.0001
Bronchoscopy	137 (37%)	10	(0–60)	0.0001
Gap measurement	112 (30%)	0	(0–27)	0.0001
Gap VB	80 (71%)	20	(10–72)	0.0001
Chromosomal investigation	112 (30%)	22	(10–46)	0.0001
Esophagoscopy	28 (8%)	0	(0–0)	0.0001

**Table 4:** Correlation between number of patients entered in the Registry and prevalence of the diagnostic investigations in the participating Institutions (Institutions with <3 patients entered in the Registry were excluded from this analysis).

**Diagnostic study**	**Pearson *r***	***p***
Abdominal ultrasound	−0.07	0.77
Head ultrasound	−0.37	0.10
Abdominal X-rays	−0.26	0.25
Skeletal X-rays	−0.12	0.60
Bronchoscopy	0.13	0.56
Oesophagoscopy	0.36	0.11
Gap measurement	0.10	0.67
Chromosomal test	0.03	0.89

## Discussion

In an analysis of over 370 neonates with EA/TEF prospectively enrolled from several pediatric surgical units participating in the EUPSA-EAR, we found a significant inter-institutional and intra-institutional heterogeneity in patient diagnostic work-up.

Previous multicenter studies on EA/TEF management have focused only marginally on the diagnostic work-up and have not described the differences in approaches among the participating centers (2–8). Significant differences in procedures among centers may lead to difficulties in benchmarking centers and comparing results. In the present study, specifically dedicated to the diagnostic work-up of patients with EA/TEF, we found a significant variability of the diagnostic approaches between the participating centers. The variability was not correlated to the number of patients registered by the different Institutions. Also, the rarest forms of EA (type B, D, and E according to Gross classification (9)), did not seem to have a more homogeneous diagnostic work-up as compared to the more frequent forms. Only chest X-rays and echocardiogram were performed in almost every patient in all participating center. All the other examinations were performed with a variable prevalence among the participating centers (Table 3, Figures 1, 2). Previous surveys found that the diagnostic approach to patients with EA/TEF was not uniform among the surveyed surgeons. In a survey of pediatric surgeons attending the joint BAPS-EUPSA meeting in Rome in 2012, Zani et al. (5) report that 81% of respondents would request a preoperative echocardiogram (while only 56% would approach the EA/TEF from the left side should a right aortic arch be detected) and 43% would perform a pre-operative bronchoscopy. In the same survey, the gap was reported to be measured intraoperatively by 60% of the respondents and routine esophagoscopy to be performed by less than 20% of respondents. Other diagnostic procedures included in our study were not surveyed. Our findings confirm the ones from Zani et al., but at the Institutional level. Reusens et al. (4), reviewing the policies of pediatric surgical units in Belgium found that preoperative bronchoscopy was performed in 36 and 46% of the Units, depending on the presence or absence of a TEF. In present cohort of patients, only 14% of patients without a TEF had a bronchoscopy, that is much lower than what reported by Reusens et al. Conversely, the prevalence of bronchoscopy in patients with a TEF in the two studies was similar (38 and 36%). Intraoperative gap assessment was reported by 43% of the units. Routine esophagoscopy was performed in 21% of the units in case of EA with TEF and 31% of the units in case of EA without TEF. These surveys were not specifically designed to detect variability in diagnostic approaches. Therefore, it is not possible to infer from these data on the variability among different Institutions. Lal et al. (2), reported on the perioperative management of EA/TEF patients treated by the centers participating in the Midwest Pediatric Surgery Consortium between 2009 and 2014. They found wide variations in the use of several identified practices, including some diagnostic examinations such as bronchoscopy, which was performed in 64% of the patients, with a range from 0 to 100% among the participating hospitals. From these data and our findings, it is clear that wide variability in diagnostic practices exists between different Institutions. This inter-Institution variability may depend on the different case mixes among the Institutions or may be secondary to different protocols used. Whatever the reason, this variability may lead to difficulties in the comparison of different Institutions and series in terms of patient populations, practices and results.

Our study and the one by Lal et al. (2), also suggest a huge intra-Institutional variability in the diagnostic work-up, that does not seem to be associated with the number of patients treated by the Institutions. In a single institution, most of the identified examinations are not performed in all patients, but only in a minority of them. These data suggest the lack of strictly defined protocols guiding the diagnostic examinations of EA/TEF patients, although such protocols are considered crucial in the different phases of management, from surgical planning to long-term follow-up (10–12). This is particularly true in some categories of patients with EA/TEF, including extremely low birth weight infants, patients with a long gap, with severe associated anomalies, or with a previously failed anastomosis, that may represent difficult clinical challenges for the surgeon. Therefore, a well-defined investigative strategy may help to drive and prioritize surgical management (11, 12) and follow-up program. However, there are a paucity of guidelines for the management EA/TEF. The difficulty in developing sound evidence-based guidelines may be due, at least in part, to the rarity of EA/TEF. To understand the magnitude of the problem, if one considers that EA/TEF incidence is roughly 1 in 4,000 live births (13, 14), based on 2018 birth rates (15) ~1,100 neonates with EA would be expected in 28-country Europe. Furthermore, considering that there are around 450 European pediatric surgery units (16), an average of less than 3 patients would be seen in each center every year. It is clear that no single institution will have a sufficient number of children to design powerful enough studies to provide accurate and meaningful evidence. The logical step would be to design national and international registries that may be instrumental in centralizing and collecting data to give an overall picture of the disease, which could be the basis for guidelines formation and subsequent prospective studies and randomized trials, and provide quality indicators that may be used to determine hospitals' performance and best practice variations. The importance of pediatric collaborative networks for the progress in clinical care of patients with rare disorders has been already pointed out (17–20). Accordingly, the European Union is currently making an effort to develop reference networks (ERN) of expert institutions for the treatment of rare disorders, among whom ERNICA will focus specifically on congenital gastro-intestinal disorders, including EA/TEF^1^. The INoEA (International Network on Esophageal Atresia) is another international collaboration dedicated to improving research and care for EA patients, which has the development of a collaborative database as one of its goals (18). The development of these collaborations is currently underway. At present, few registries are specifically dedicated to, and gather data on, EA/TEF patients: the French National Esophageal Atresia Register (21), the Turkish Esophageal Atresia Registry (22), and the EUPSA-EAR, which is the only one at an international level. Whilst waiting for the development of ERNICA and INoEA registries, the data from the existing registries may prove helpful in advancing EA/TEF patient care. In addition, these registries should continue recording data, also to allow comparisons with new developing registries data in the future.

This study has some limitations. Firstly, it is possible that not all patients treated by each Institution were entered in the Registry, as the Registry has a voluntary basis. This may lead to biased analysis, as some patients may be missed from the analysis. However, it is plausible that the inclusion of more patients would increase rather than reduce the variability in diagnostic work-up. Second, the list of potential diagnostic procedures is not exhaustive. However, the analysis of the diagnostic procedures included in the study gives an idea of the extreme variability that exists between different centers in the diagnostic work-up of EA/TEF patients. Finally, the data collection was not purposely designed to detect differences in EA/TEF diagnostic work-up. As a result, some data may be missing or not declared, and this potential bias may lead to an overestimation of the differences between centers.

It goes beyond the scope of this study to discuss the correct diagnostic work-up for patients with EA/TEF, although a thorough preoperative work-up may change significantly the management of a patients with EA/TEF. For example, a preoperative tracheobronchoscopy may allow to change the diagnosis from Gross type A EA (without TEF) to type B EA (with a proximal TEF), that modifies significantly the treatment and avoids leaving behind an unknown proximal TEF (23).

In conclusion, this first study from the first international registry on EA/TEF on a relevant number of patients shows inter-institutional and intra-institutional variability in the diagnostic work-up of patients with EA/TEF. Efforts should be made to develop shared guidelines on diagnostic workup for EA/TEF patients. The use of currently available registries may help in gathering data and promote the development of such guidelines.

## Data Availability Statement

The raw data supporting the conclusions of this article will be made available by the authors, without undue reservation, to any qualified researcher.

## Ethics Statement

The EUPSA-EAR is based in the University of Graz and has been approved for use by its Ethical Committee (number 27-259 ex 14/15). Individual research studies using EUPSA-EAR data do not require further separate ethical approval, if only anonymized data are used.

## Author's Note

Members of the EUPSA Esophageal Atresia Registry who contributed patients and data between July 2014 and July 2017:

FM, PB, AC, Ospedale Pediatrico Bambino Gesù, IRCCS, Rome, Italy.
Helen Engstrand Lilja, University Children's Hospital, Uppsala, Sweden.
Henrik Ehren, Karolinska University Hospital, Stockholm, Sweden.
Udo Rolle, Department of Pediatric Surgery and Pediatric Urology, Johann Wolfgang-Goethe University, Frankfurt, Germany.
Soyer Tuktu, Department of Pediatric Surgery, Hacettepe University, Ankara, Turkey.
Zoran Radojicic, Marija Lukac, University Children's Hospital, Belgrade, Serbia.
EH, Matthijs Oomen, Pediatric Surgical Academic Medical Center, Amsterdam, The Netherlands.
Pier Giorgio Gamba, Francesco Fascetti Leon, Pediatric Surgery Unit, University of Padua, Padua, Italy.
Ivo de Blaauw, Horst Scharbatke, Radboud UMC—Amalia Children's Hospital, Nijmegen, The Netherlands.
Rene Wijnen, Sophia Children's Hospital Erasmus MC, Rotterdam, The Netherlands.
Holger Till, FF, Medical University of Graz, Graz, Austria.
Lubomir Bockanic, Michel Gocik, Children Teaching Hospital, Košice, Slovacchia.
Sergey V. Minaev, Department of Pediatric Surgery, Stavropol State Medical University, Stavropol, Russia.
Liviu Muntean, Aurel Mironescu, Brasov Children's Hospital, Brasov, Romania.
Risto Rintala, Helsinki University Central Hospital, Helsinki, Finland.
Hussein Khairy, Mahmoud ElFiky, Cairo University Children's Hospital, Cairo, Egypt.
Giovanna Riccipetitoni, Sara Costanzo, Vittore Buzzi Children's Hospital, Milan, Italy.
Pierluigi Lelli Chiesa, Gabriele Lisi, University "G d'Annunzio Chieti-Pescara, Italy.
Juan A. Tovar, Leo Martinez, Hospital Universitario La Paz, Madrid, Spain.
Alessio Pini Prato, Germana Casaccia, Alessandria Children's Hospital, Alessandria, Italy.
Benno Ure, Joachim Kuebler, Hannover Medical School, Hannover, Germany.
Jiri Snajdauf, Second Faculty of Medicine, Charles University, Prague, Czech Republic.
AZ, Royal London Hospital, London, United Kingdom.

## Author Contributions

FM analyzed the data drafted the manuscript and approved the final version of the manuscript. All authors contributed to the article and approved the submitted version.

## Conflict of Interest

The authors declare that the research was conducted in the absence of any commercial or financial relationships that could be construed as a potential conflict of interest.

## Footnotes

^1^Available online at: https://ec.europa.eu/health/sites/health/files/ern/docs/ernica_factsheet_en.pdf
